# Cracks-YOLO: an improved YOLOv5 tongue crack detection method based on switchable atrous convolution and transformer

**DOI:** 10.3389/fmed.2026.1901533

**Published:** 2026-09-29

**Authors:** Miaomiao Ding, Meiyi Wu, Bochen Shen, Huangbo Lin, Shaoyang Men

**Affiliations:** 1 School of Medical Information Engineering, Guangzhou University of Chinese Medicine, Guangzhou, China; 2 School of Business, City University of Hong Kong, Kowloon, Hong Kong SAR, China; 3 School of Biomedical Engineering, Tsinghua University, Beijing, China

**Keywords:** cracks detection, switchable atrous convolution, tongue diagnosis, transformer encoder, YOLOv5

## Abstract

**Introduction:**

Tongue diagnosis is a vital component of Traditional Chinese Medicine (TCM), with cracked tongue serving as a key diagnostic indicator. Although deep learning techniques have advanced TCM tongue image recognition, existing methods for cracked tongue detection still face challenges of low accuracy and slow processing speed.

**Methods:**

To address these issues, we propose an improved YOLOv5 framework integrating Switchable Atrous Convolution (SAC) and a Transformer encoder. First, given the varied morphology, size, and distribution of tongue cracks, SAC is introduced into the backbone network to adaptively adjust receptive fields via different atrous rates, enabling multi-scale feature extraction and better preservation of fine details. Second, a Transformer encoder module is incorporated to capture global information and long-range dependencies, thereby enhancing the network's overall feature representation capability. Experiments are conducted on a self-built tongue image dataset to evaluate the proposed modifications.

**Results:**

The improved YOLOv5 demonstrates superior performance over the original YOLOv5 in tongue crack detection, achieving an increase of 2.2% in AP@50 and 8.43% in AP@50:95 metrics on the self-built dataset.

**Discussion:**

These results confirm that the combination of SAC and Transformer effectively boosts detection accuracy and generalizability, addressing the limitations of prior approaches. The proposed method offers a more reliable and efficient solution for automated tongue crack analysis, with potential implications for clinical TCM diagnostic support.

## 1 Introduction

Tongue diagnosis is an essential component in Traditional Chinese Medicine (TCM) diagnosis. Within the framework of TCM theory, the examination of the tongue, which includes the color and morphology of the tongue body, is indicative of the clinical significance of the body’s zang-fu organs, meridians, qi, blood, fluid and bodily fluids. By grasping the patterns within tongue diagnosis, doctors can determine the patient’s constitution and thereby differentiate the syndrome and its appropriate treatment. Among the various tongue manifestation, a fissured tongue is a significant diagnostic indicator, characterized by the presence of cracks on the tongue surface without the coverage of a tongue coating (1). Lin et al. (2) proposed MetaLink, an imbalanced multi-label classification framework for automatic tongue diagnosis, demonstrating that intelligent tongue analysis account for the coexistence of multiple tongue manifestations and their inherently skewed clinical distributions. Numerous studies have demonstrated a close relationship between tongue manifestation and major diseases such as diabetes, coronary heart disease and fatty liver (3–7). Nevertheless, traditional methods of tongue diagnosis are inevitably limited by factors such as the clinician’s experience and the diagnostic environment. Consequently, the pursuit of objectification of tongue diagnosis has become particularly crucial and urgent. With the advancement of information technology, techniques such as image processing and machine learning have progressively matured in medical image processing. Ju et al. (8) developed a network for liver tumor segmentation that explicitly models scale and boundary ambiguities in irregular targets. Ju et al. (9) proposed a collaborative segmentation strategy fusing dynamic and static information, suggesting complementary feature modeling improves structural representation in complex biomedical images. Similar medical image processing techniques have also found application in the objectification of tongue diagnosis (10). Sun et al. (11) proposed a methodology and conceptual framework for the qualitative and quantitative analysis of tongue manifestation based on image technology, which marked a significant exploration in the field of objective tongue diagnosis. Yu et al. (12) employed a fuzzy clustering method on the RGB values of tongue manifestation to define the domain of tongue characteristics, facilitating the classification of tongue manifestation. Zhu et al. (13) segmented the tongue manifestation into multiple feature blocks and utilized K-Means clustering analysis based on the CIE color space model to analyze the color features of each segment, as well as Gabor filters to capture color contrast features and linear judgment functions to analyze the texture characteristics of the tongue body. Xu et al. (14) delineated the regions of tongue manifestation using a split-merge algorithm and chroma threshold method, achieving the separation of tongue substance and tongue coating. Srividhya et al. (15) have proposed a computer-aided diagnostic system predicated on the analysis of tongue imagery. This system employs image segmentation and feature extraction techniques, in conjunction with a self-organizing map classifier, with the objective of enhancing the precision and efficiency in the diagnosis of diabetes mellitus. The aforementioned methodologies leverage intuitive and comprehensible image processing and analysis techniques, offering the advantages of strong interpretability and ease of training. However, these approaches encounter significant challenges when applied to tasks of increased complexity.

To mitigate such challenges, Ju et al. (16) improved small intestinal stromal tumor detection by incorporating 3D context and auxiliary prior cues, indicating that contextual dependency and priorguided representation are crucial for detecting subtle and spatially complex lesions. This successful strategy has inspired growing interest in the objectification of tongue diagnosis, where deep learning techniques have demonstrated remarkable performance, as noted by Wu et al. (17). In a seminal work, Li et al. (18) proposed a novel Local Correlation Loss (LCLoss) function, predicated on regional correlation, which they adeptly applied to the segmentation of the tongue body. The efficacy of this innovative approach was empirically corroborated through its application in U-Net and Fully Convolutional Network (FCN) models. Subsequently, they architected an analytical model that synthesizes geometric and textural features of tongue manifestation on the foundation of the VGG16 network, facilitating the classification of various tongue manifestations, including those with fissures. Nonetheless, this methodology, while effective in discerning the presence of fissured tongues, is constrained in its ability to provide detailed spatial information. Xue et al. (19) proposed a technique to extract depth features of fissures leveraging the AlexNet architecture and employed a Multiple-Instance Support Vector Machine (MI-SVM) for the identification of tongue manifestations. However, akin to the aforementioned approach, this method also falls short in furnishing precise locational data. Chen et al. (20) introduced an enhanced version of the Faster Region-based Convolutional Neural Network (Faster R-CNN) for the detection of fissures on the tongue surface. This technique utilizes the ResNet-50 within a Region Proposal Network (RPN) to retain a greater wealth of original image data and incorporates an attention mechanism to accentuate fissure information. Despite its innovative contributions, the method’s complexity and computational expense are considerable, posing challenges in meeting practical application demands. Tang et al. (21) introduced an expansion encoder network (DE-Net) designed to capture a plethora of advanced features, thereby achieving high-resolution outputs for the purpose of automated tongue image segmentation. Weng et al. (22) proposed a Weakly Supervised YOLO (WS-YOLO) method, grounded in the Darknet-53 network. This method employs tongue images with complete bounding box annotations and coarse image-level annotations to train models for the detection of teeth marks and fissures, significantly alleviating the burden of annotation work for such tongue presentations. However, there remains a pressing need to augment the detection precision of this method to ensure its reliability and effectiveness in clinical and research settings.

The YOLOv5 algorithm (23) is a quintessential one-stage object detection framework, renowned for its exemplary balance between detection velocity and accuracy, which has led to its widespread adoption across various domains. YOLOv5 employs the lightweight feature extraction network CSP-Darknet53 as its backbone, which is characterized by its high efficiency and commendable feature representation capabilities. However, given the intrinsic characteristics of tongue surface fissures, which often manifest as varied morphologies and uneven distributions, the application of YOLOv5 in the task of tongue fissure detection may be susceptible to the loss of detailed information pertaining to fissure features during the extraction and fusion phases. This, in turn, can lead to a diminution in the precision of the detection outcomes.

In the present investigation, we have developed a novel approach to tongue crack detection, which is an extension of the YOLOv5 framework. Our contributions are delineated as follows:

- Enhancement of the YOLOv5 architecture through the integration of Switchable Atrous Convolution (SAC) in the backbone network, enabling adaptive atrous rate adjustment for improved multi-scale representational capacity and enhanced fissure information extraction.
- Augmentation of the network’s capabilities through the integration of a Transformer encoder module, facilitating the modeling of global context and long-range dependencies for comprehensive semantic information capture, leading to increased detection accuracy and robustness.
- Experimental validation on a self-constructed dataset showcasing a notable 2.2% improvement in the AP@50 metric and an 8.43% improvement in the AP@50:95 metric compared to the original YOLOv5, indicating enhanced detection precision.
- Achieving a high detection velocity of 139 frames per second in the experimental environment, with a modest computational overhead, demonstrating practical feasibility for real-world applications.

## 2 Methods

The YOLOv5 framework encompasses five distinct versions: YOLOv5n, YOLOv5s, YOLOv5m, YOLOv5l, and YOLOv5x, each corresponding to varying degrees of network depth and feature map dimensions, escalating in size. The YOLOv5 suite comprises five variations—YOLOv5n, YOLOv5s, YOLOv5m, YOLOv5l, and YOLOv5x—that correspond to a spectrum of network depths and feature map dimensions, escalating in scale from the smallest to the largest. The backbone of these models is the lightweight feature extraction network CSP-Darknet53, which is anchored in the Darknet53 architecture (24). This backbone incorporates the strategy of CSPNet (25), entailing the bifurcation of feature maps into two distinct subsets at each stage, with subsequent merging across hierarchical layers. This design augments gradient propagation within the network, thereby enhancing its learning efficacy and simultaneously alleviating computational constraints and diminishing memory consumption. Nonetheless, the strategies employed during the feature extraction phase may precipitate the loss and conflation of information within the feature maps, leading to the erosion of detailed information and consequently compromising the accuracy of the model in the detection of tongue surface fissures.

In response to these challenges, the current study introduces an augmented YOLOv5 architecture based on the YOLOv5s configuration, which integrates SAC and Transformer modules. This enhancement, as depicted in Figure 1, is crafted to preserve and accentuate the finer nuances within the feature maps, thereby augmenting the model’s precision in the detection of tongue cracks.

**Figure 1:**
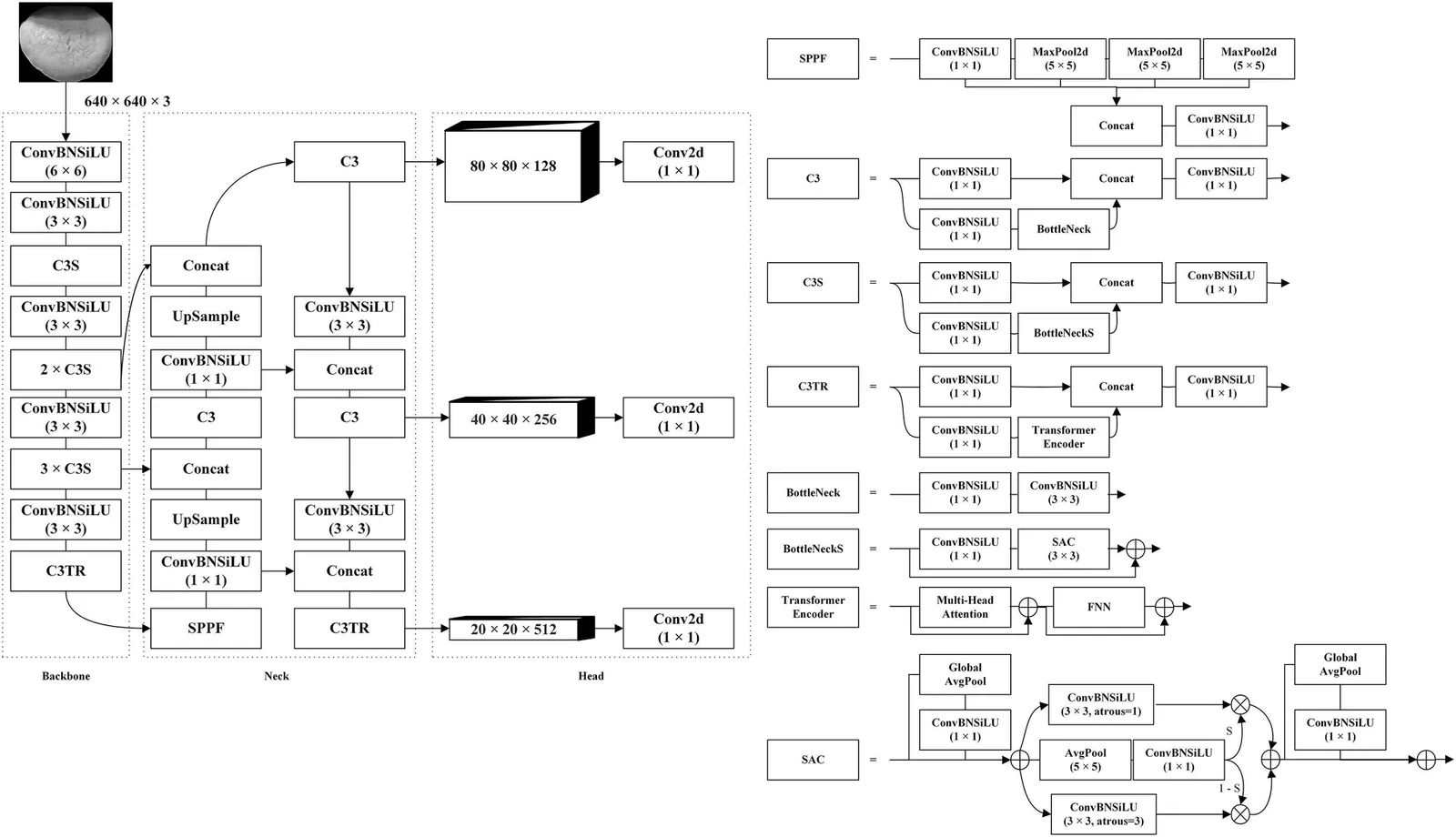
Improved YOLOv5 network structure based on Switchable Atrous Convolution and Transformer.

In this study, we have augmented the YOLOv5s network architecture by integrating Switchable Atrous Convolution and Transformer encoders within the backbone and neck of the YOLOv5 framework, thereby enabling their participation in feature extraction and fusion. The SAC modulate the receptive field size of the convolutional layers by employing varying atrous rates, allowing the network to focus on features at different scales and consequently preserving detailed information more effectively. The Transformer encoder, on the other hand, constructs a global image representation through its multi-head self-attention mechanism. This facilitates the network’s ability to capture long-range dependencies and global contextual information more adeptly, thereby providing more precise detail information. The synergistic application of these advanced techniques within the YOLOv5 architecture is anticipated to significantly enhance the model’s performance in detecting intricate features such as tongue surface fissures.

### 2.1 Improved network based on switchable atrous convolution

Atrous Convolution (26, 27), also known as Dilated Convolution, is an efficacious convolutional technique that serves to augment the receptive field of a network without incurring the addition of extraneous parameters. This is achieved by modulating the atrous rate or dilated rate, which controls the size of the receptive field, thereby enabling the network to detect objects at varying scales whilst employing identical convolutional weights. Let $F\;:\;Z^{2}\to R$ denote a discrete function, and define $\Omega_{r}=[-r,r{]}^{2}\cap Z^{2}$ as the domain of the function. Furthermore, let $k\;:\;\Omega_{r}\to R$ be a discrete convolution kernel of size $(2r+1{)}^{2}$. The specific process of atrous convolution unfolds as follows:

$$\;\left(F\;{*}_{\ell }\;k\right)\left(\;p\right)=\sum_{s+\ell t=p}F\left(s\right)k\left(t\right)$$

Within this context, the notation ${*}_{\ell }$ signifies an atrous convolution or, more specifically, an ℓ-Dilated Convolution. When the atrous rate ℓ=1, * is tantamount to the ordinary discrete convolution. That is to say, this convolutional operation achieves the effect of enlarging the network’s receptive field without incurring additional parameters by selectively weighing and summing elements within the convolutional kernel through a skipping mechanism, as illustrated in Figure 2: In the realm of convolutional computation, (a) when the atrous rate l=1, the receptive field size is 3×3; (b) when the atrous rate l=2, the receptive field expands to 7×7; (c) and when the dilation rate l=4, the receptive field encompasses a size of 15×15. Consequently, atrous convolution has garnered widespread application in recent years due to its capacity to modulate the network’s receptive field by adjusting the atrous rate without the addition of extraneous parameters, as evidenced in applications such as image super-resolution (28), multi-scale feature extraction (29) and a plethora of other tasks within the domain of computer vision.

**Figure 2:**
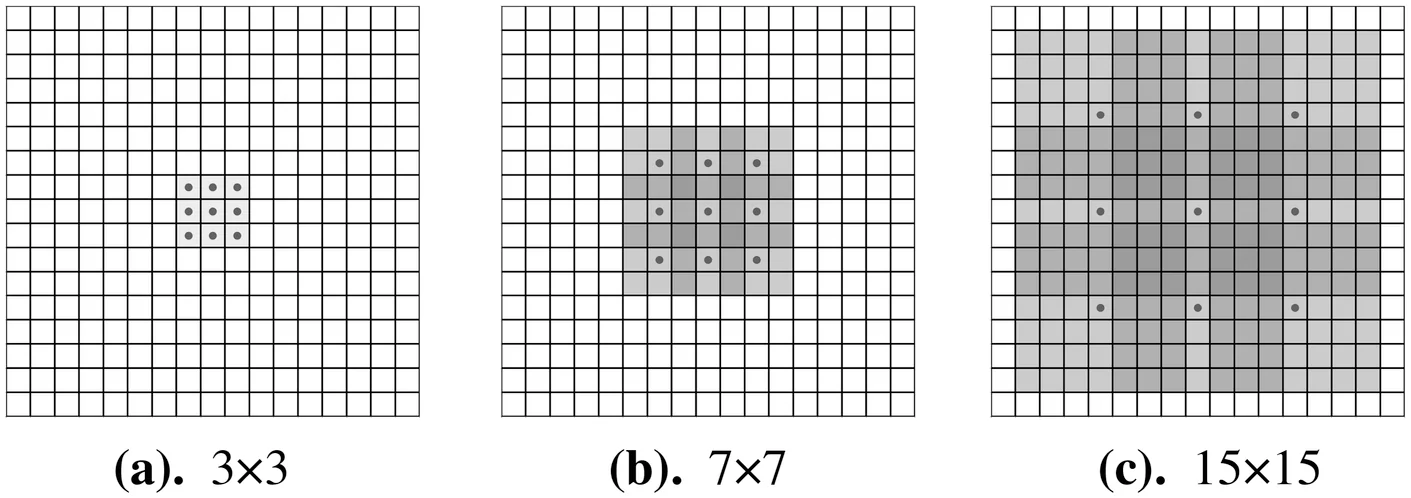
Atrous convolution illustration. **(a)** 3×3 **(b)** 7×7 **(c)** 15×15.

Based on atrous convolution, the Switchable Atrous Convolution (SAC) (30) facilitates dynamic transitions between disparate dilation rates, denoted as ℓ and amalgamates the convolutional outcomes associated with distinct dilation rates using switch functions. and amalgamates the convolutional outcomes associated with distinct dilation rates using switch functions. Conv(x,w,ℓ) , where x is the input, w represents the weights, and ℓ signifies the atrous rate, is articulated as follows:

$$\;\begin{array}{rl} & Conv(x,w,1)\Rightarrow S\left(x\right)∙Conv(x,w,1)\\ & \;+[1-S(x)]∙Conv(x,w+\Delta w,l) \end{array}$$

In this framework, Δw represents the trainable weights, while the atrous rate ℓ is considered a hyperparameter, which is set to 3 in the context of this study. The switch function, denoted as S(∙) is realized as a concatenated sequence of a 5×5 average pooling layer followed by a 1×1 convolutional layer, as depicted in Figure 3. This function exhibits spatial correlation, meaning that different regions of the feature map may be subject to distinct switch function controls, thereby enabling the network to effectively integrate tongue surface fissure feature information across varying scales. Consequently, this enhances the model’s performance in multi-scale tongue cracks detection tasks.

**Figure 3:**
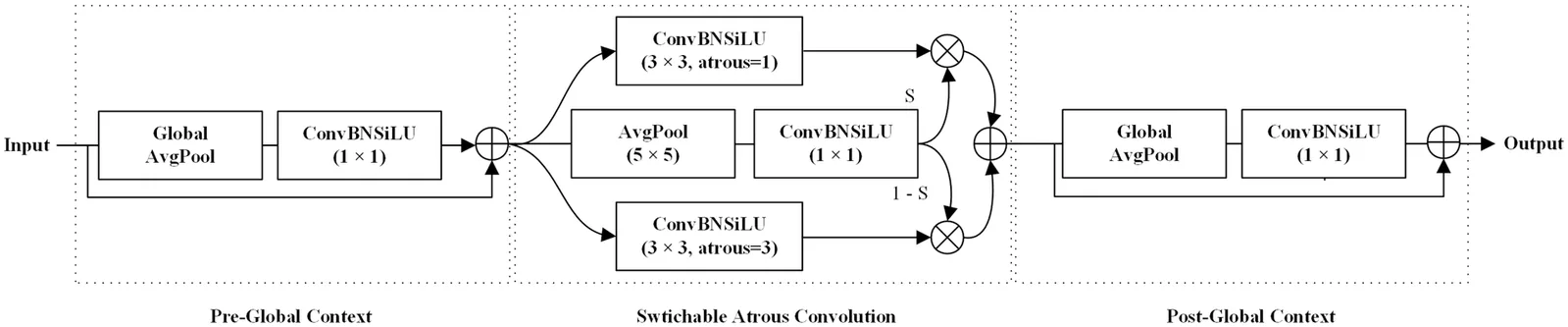
Switchable atrous convolution structure.

The architecture of the SAC is delineated in Figure 3, comprising three integral components: the Pre-Global Context module, the SAC module, and the Post-Global Context module. The SAC adeptly harnesses two lightweight Global Context modules to incorporate global information into the switch function via global average pooling. This strategic integration endows the switch function with the capability to generate more stable switching predictions, thereby augmenting the performance of tongue cracks detection.

Within the YOLOv5 architecture, CSP-Darknet53 incorporates the Cross Stage Partial (CSP) structure, which effectively partitions the gradient flow, thereby augmenting the flow of information and propagation of gradients throughout the network, This design strategy effectively mitigates computational bottlenecks and enhances the network’s learning capabilities. The CSP structure is specifically implemented through the C3 module, which operates by bifurcating the feature maps into two distinct subsets. Each subset undergoes a separate convolutional operation, followed by a concatenation step to merge the modified feature maps back into a unified representation.

To enhance the network’s capacity for multi-scale feature representation, this study introduces a SAC3 module based on Switchable Atrous Convolution, This module employs a Residual Structure (31) and a CSP configuration to replace some of the C3 modules within the CSP-Darknet53 architecture, as illustrated in Figure 4. Specifically, for an input feature map $X\in R^{H\times W\times C}$, it is initially bifurcated into two pathways, designated as Pathway One and Pathway Two. In Pathway One, the feature map undergoes a 1×1 convolutional layer to yield a new feature map $X_{1}\in R^{H\times W\times C_{1}}$, Pathway Two processes the feature map through a 1×1 convolutional layer followed by a residual structure that integrates SAC. This structure comprises N residual–connected 1×1 convolutional layers and 3×3 SAC layers. The residual structure sums the original input of the residual block with the output of the convolutional operation, thereby generating a novel feature map $X_{2}\in R^{H\times W\times C_{2}}$. The two feature maps, $X_{1}$ and $X_{2}$ are then concatenated to form $X_{\mathrm{concat}}\in R^{H\times W\times (C_{1}+C_{2})}$, which, after passing through a 1×1 convolutional layer, results in the final output $X_{\mathrm{out}}\in R^{H\times W\times C}$. Throughout this process, Batch Normalization (32) is applied to bolster the representational power of the features and to enhance the stability of the model during training. Additionally, the Sigmoid Linear Unit (SiLU) (33) activation function is utilized to augment the non-linear expressive capabilities of the features. These methodological choices collectively contribute to a more dynamic and nuanced feature extraction process, which is essential for the accurate detection of tongue cracks across varying scales.

**Figure 4:**
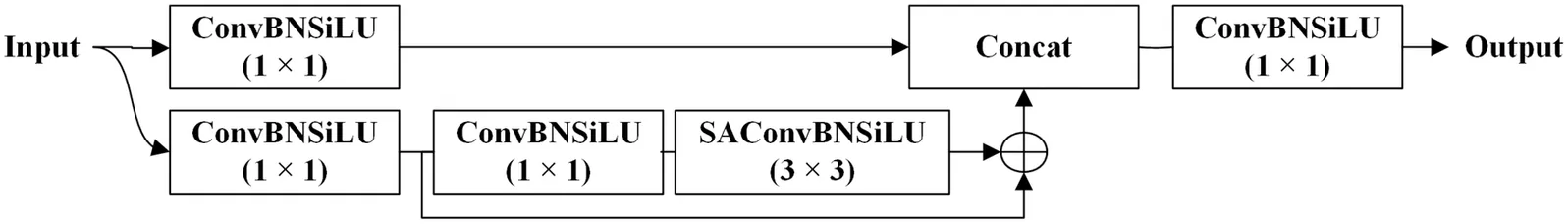
C3S network structure.

### 2.2 Improved network based on transformer encoder

Vision Transformer (ViT) (34) is a pioneering image classification model that harnesses the power of Transformers for the realm of computer vision, predicated on the Transformer encoder for the encoding and representation of image features to model the global contextual relationships within an image. The Transformer encoder architecture employed in this study is depicted in Figure 5, which is composed of Multi-Head Self-Attention (MHSA) (35) layers and Feed-Forward Neural Networks (FFN). Residual connections are judiciously utilized between the sublayers to facilitate the propagation of gradients and to mitigate the vanishing gradient issue, thereby enhancing the model’s learning efficiency.

**Figure 5:**
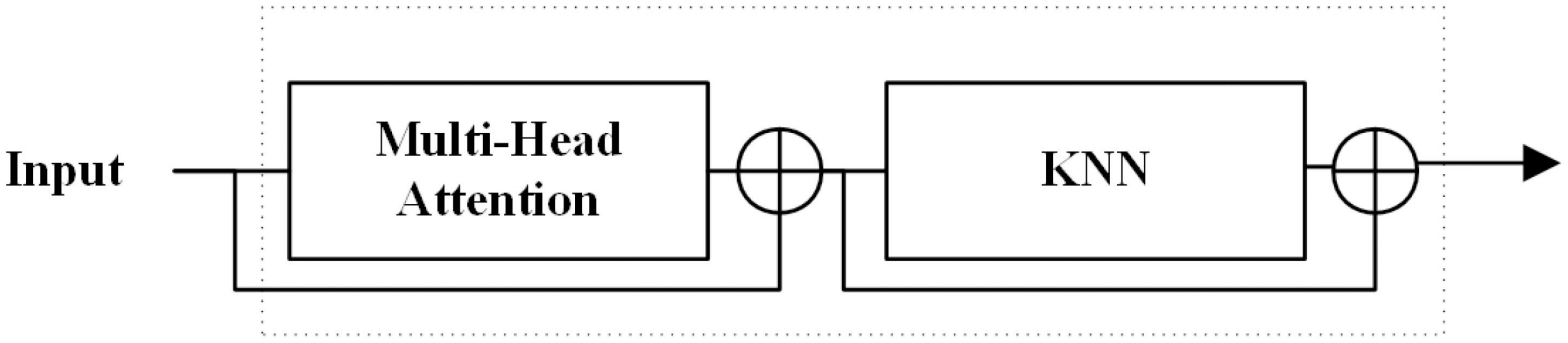
Transformer encoder structure.

The core of the Transformer encoder lies in the Multi-Head Attention (MHA) mechanism. This sophisticated attention scheme is an extension of the traditional attention mechanism, which concurrently computes multiple instances of Scaled Dot-Product Attention heads. By doing so, it enables the network to learn a variety of distinct attention weight distributions in parallel, thereby augmenting the model’s capacity to focus on and represent different locations within the tongue surface with greater acuity. The specific process unfolds as follows:

$$\;Attention(Q,K,V)=softmax\left(\frac{QK^{T}}{\sqrt{d_{k}}}\right)V$$

The Attention mechanism function, denoted as Attention(∙) meticulously computes the correlation between the query matrix Q and the key matrix K transforming these correlations into a set of weights through the softmax function. Subsequently, these weights are employed to modulate the feature matrix V culminating in the generation of the Attention mechanism’s output.

$$\;\begin{array}{cc} MHA\left(Q,K,V\right) & =\;Concat\left(head_{1},\ldots ,head_{h}\right)W^{O}\\ \;\;\;\;\;\;\;\;\;\;\;\;\;\;\;\;head_{i} & \;\;=\;\;Attention\left(QW_{i}^{Q},KW_{i}^{K},VW_{i}^{V}\right) \end{array}$$

The Multi-Head Attention mechanism function MHA(∙) operates by employing a weighted concatenation of the outputs of the Attention(∙) function, which have been transformed through a weight matrix W. This process yields the final output of the MHA mechanism. By leveraging this approach, the MHA function is adept at capturing a diverse array of features that are emphasized by different attention heads, leading to an enriched representation of the feature maps. This enhanced representational capacity enables the network to effectively model long-range dependencies and global contextual relationships, which are critical for discerning the subtleties of tongue cracks.

The neck network architecture of YOLOv5 predominantly employs the CSP-PAN structure, which is based on PANet (36, 37). As depicted in Figure 6, this architecture orchestrates an interplay between low-level, fine-grained features and high-level, semantic features through upsampling and downsampling operations, facilitating the transmission and aggregation of information across different network strata. This multi-tiered feature fusion enhances the model’s representational prowess with respect to objects of interest, thereby bolstering the precision of object localization and classification.

**Figure 6:**
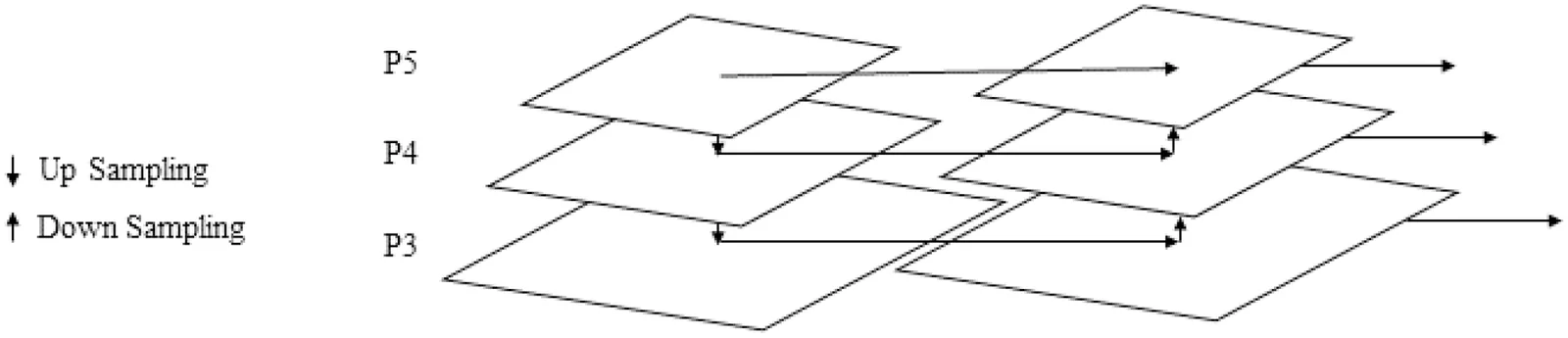
CSP-PAN illustration.

The CSP-PAN architecture, an enhancement of the PANet foundation, incorporates the CSP structure, specifically the C3 module, to supplant the conventional convolutional layers and apply a series of convolutional operations across feature maps at disparate hierarchical levels. This integration of the CSP framework is distinct from the C3 modules found within the CSP-Darknet53 architecture. Unlike their counterparts, the C3 modules implemented within CSP-PAN forgo the use of residual structures, thereby preempting the propagation of redundant information and mitigating the potential for repetitive feature dissemination.

In this paper, we have architected the C3TR module, which integrates a Transformer encoder within the CSP framework, to supplant certain C3 modules in deep neural networks, as depicted in Figure 7. Specifically, the C3TR module adheres to the CSP strategy by partitioning the input feature map $X\in R^{H\times W\times C}$ into two distinct pathways, referred to as Pathway One and Pathway Two. Pathway Two’s feature map undergoes a 1×1 convolutional layer prior to being fed into the Transformer module. Within this module, the feature map is subjected to linear transformations to derive the query (Q), key (K), and value (V) matrices, which are then utilized in the MHSA layer for attention computation. The outcomes are subsequently aggregated with the original input through an addition operation. The feature map is then passed through a feed-forward neural network, composed of two fully connected layers, to undergo non-linear transformations, and is again combined with the input through an addition operation, yielding the feature map $X_{2}\in R^{H\times W\times C_{2}}$, Ultimately, the feature map $X_{1}\;$ from Pathway One, which has undergone a single 1×1 convolution, is concatenated with $X_{2}$ to form $X_{\mathrm{concat}}\in R^{H\times W\times (C_{1}+C_{2})}$, After traversing a final 1×1 convolutional layer, the output $X_{\mathrm{out}}\in R^{H\times W\times C}$.

**Figure 7:**
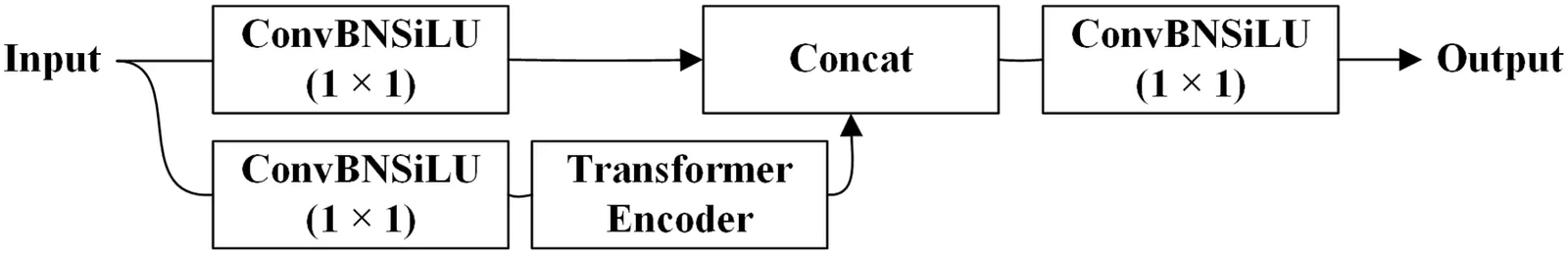
C3TR network structure.

Theoretically, the Transformer architecture is posited to possess superior expressive capabilities compared to traditional convolutional neural networks (CNNs). However, due to the high computational complexity associated with the global attention mechanism, particularly when the feature map dimensions are large in shallower networks, the Transformer can incur substantial computational costs. Consequently, recent research endeavors (37, 38) have explored the integration of Transformers with CNNs, such as interlacing Transformer modules within the backbone network or substituting certain convolutional blocks with Transformer modules. This approach aims to enhance the network’s modeling capabilities. Inspired by these studies, this paper integrates the Transformer modules into both the backbone and the terminal C3 modules of the YOLOv5 neck network. The rationale behind this integration is that the feature map sizes at the network’s terminus are relatively smaller, which can effectively mitigate the significant computational expenses associated with the computation of global attention. Furthermore, this work streamlines the Transformer encoder structure from the ViT by eliminating the Layer Normalization layers and simplifying the Multi-Layer Perceptron (MLP) to a feed-forward neural network composed of two fully connected layers. These modifications are intended to preserve the transformative benefits of the Transformer architecture while optimizing its computation. In this study, experiments were conducted on a proprietary tongue image dataset using the YOLOv5s as the foundational network. The primary metrics for evaluating the accuracy of tongue crack detection were the Average Precision (AP) at an Intersection over Union (IoU) threshold of 0.5 (AP@50) and at a 50:95 scale (AP@50:95). Additionally, the computational complexity and efficiency of the model were assessed using, Giga Floating-Point Operations (GFLOPs), and the number of parameters.

In this study, experiments were conducted on a proprietary tongue image dataset using the YOLOv5s as the foundational network. The primary metrics for evaluating the accuracy of tongue crack detection were the Average Precision (AP) at an Intersection over Union (IoU) threshold of 0.5 (AP@50) and at a 50:95 scale (AP@50:95). Additionally, the computational complexity and efficiency of the model were assessed using, Giga Floating-Point Operations (GFLOPs), and the number of parameters.

The model presented in this paper was implemented in the PyTorch 1.12.1 environment and trained and tested on an NVIDIA GeForce RTX 2080 GPU experimental setup, with the operating system being Ubuntu 18.04. To expedite the training process, pre-trained weights from the COCO object detection dataset (39, 40) were utilized to facilitate rapid model convergence.

The model was trained for 600 epochs on the tongue image dataset, with the initial three epochs dedicated to a gentle warm-up phase. The batch size was configured as 32, and the input dimension to the network was set to 640×640 pixels. The initial learning rate was established at 0.001, with parameters updated using the Adam optimizer. The weight decay was set to 0.0005 to regularize the learning process and prevent overfitting.

### 2.3 Placement of C3S and C3TR modules

Tongue cracks vary in scale and contrast and may be confused with coating patterns or uneven illumination. Cracks-YOLO therefore replaces only selected C3 blocks with C3S and C3TR modules, rather than applying either module throughout the network.

C3S is used at $X_{3}$, $X_{5}$, and $X_{7}$, corresponding to feature-map resolutions of H/4×W/4, H/8×W/8, and H/16×W/16, respectively. Each C3S block incorporates SAC with atrous rates E={1,3} to combine local and wider-context features without further spatial downsampling. These stages retain relatively detailed spatial information, while restricting C3S to them limits the additional computation introduced by the SAC branches.

C3TR is used at $X_{9}$ and $X_{24}$, both operating at H/32×W/32. Since the computational cost of self-attention grows quadratically with the number of spatial tokens, applying C3TR only to low-resolution feature maps keeps the cost manageable. The backbone C3TR aggregates global context before SPPF, whereas the neck C3TR refines the coarse-scale feature after the final PAN fusion.

Algorithm 1 summarizes the inference path of Cracks-YOLO. The top-down FPN fuses the backbone features $X_{7}$ and $X_{5}$, while the bottom-up PAN reuses $X_{15}$ and $X_{11}$. The three detection heads operate on $X_{18}$, $X_{21}$, and $X_{24}$, corresponding to resolutions of H/8, H/16, and H/32, respectively.

Algorithm 1 **Deterministic forward propagation of cracks-YOLO.**

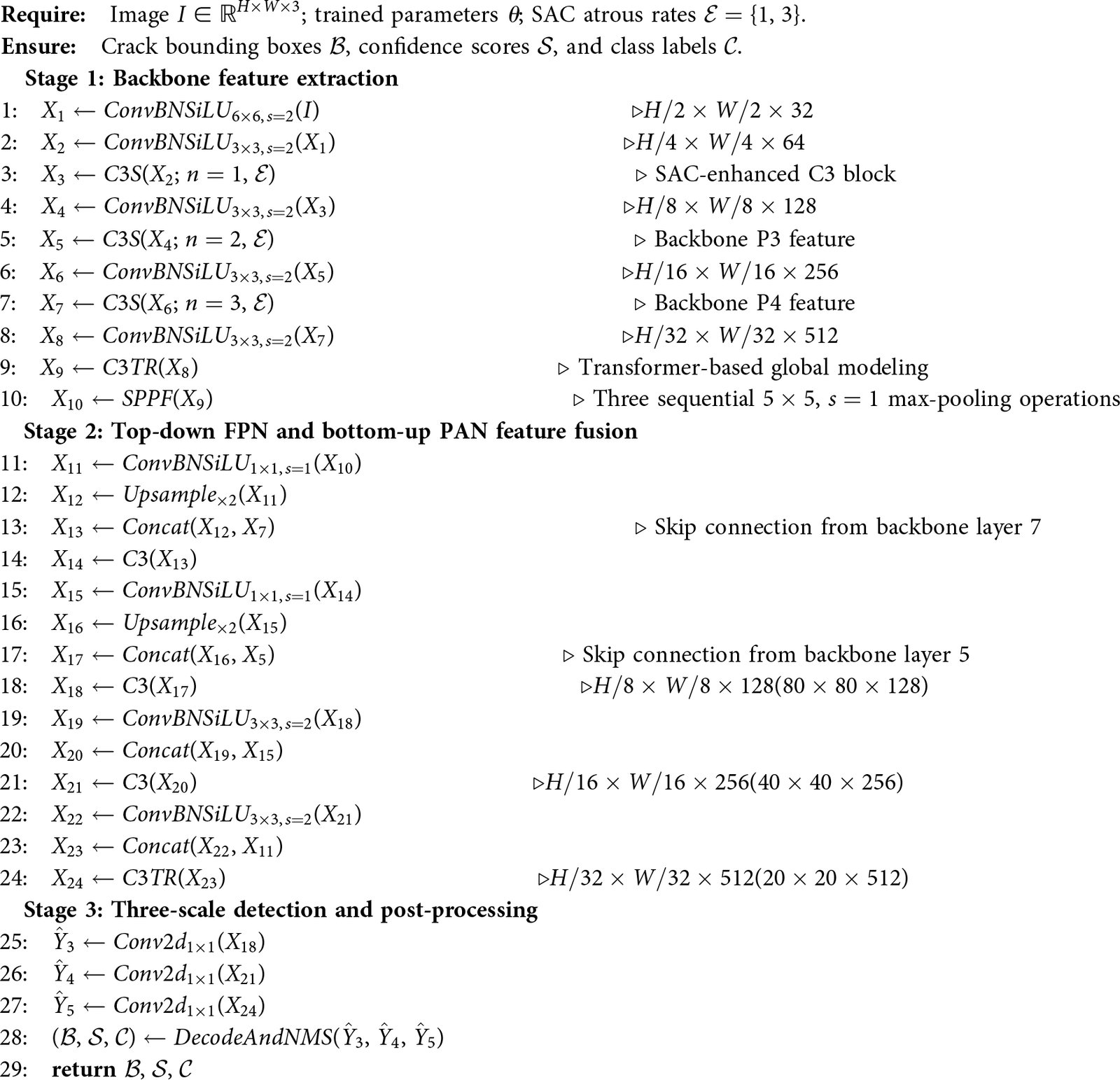

### 2.4 Dataset

The tongue image dataset utilized in this study was sourced from the Shenzhen Traditional Chinese Medicine Hospital and comprises a total of 715 tongue images. These images encompass a diverse array of tongue surface data collected under various conditions, including different imaging devices, lighting conditions, angles of photography, and environmental settings.

In light of the potential interference from redundant information external to the tongue surface in the detection of tongue cracks, this experiment leverages an instance segmentation algorithm provided by YOLOv5 to train a tongue body segmentation model. This model is employed to segment and crop the tongue surface data, thereby eliminating extraneous information and enhancing the detection performance.

The tongue image dataset, as exemplified in Figure 8, is segmented into two distinct components: tongue body segmentation and tongue crack detection, each subjected to a separate data annotation process. The annotation of tongue cracks was conducted under the supervision of specialized physicians from Guangzhou University of Chinese Medicine, following the principle of closely encircling the target cracks. The annotations targeted cracks on the tongue surface where pathological features were markedly evident and the cracks were clearly discernible. For the tongue body segmentation portion, a total of 715 tongue images were annotated with a standardized image resolution of 640×640 pixels. In the context of tongue surface crack detection, the dataset was expanded from the original set by incorporating data augmentation techniques such as image flipping and color temperature adjustment, thereby increasing the dataset to 1,100 images, all with a uniform resolution of 640×640 pixels. Comprehensive statistical details of the tongue image dataset are delineated in Table 1. The implementation of instance segmentation in YOLOv5 is informed by the principles of YOLACT (41), It incorporates a prototype network, referred to as Protonet, into the detection head of YOLOv5 to generate prototype masks. These masks are then linearly combined with mask coefficients to produce instance masks, facilitating pixel-level mask generation. This approach eschews the reliance on repooling, thereby forgoing the explicit feature localization step. Consequently, it is capable of generating high-quality masks while maintaining real-time computational velocity.

**Figure 8:**
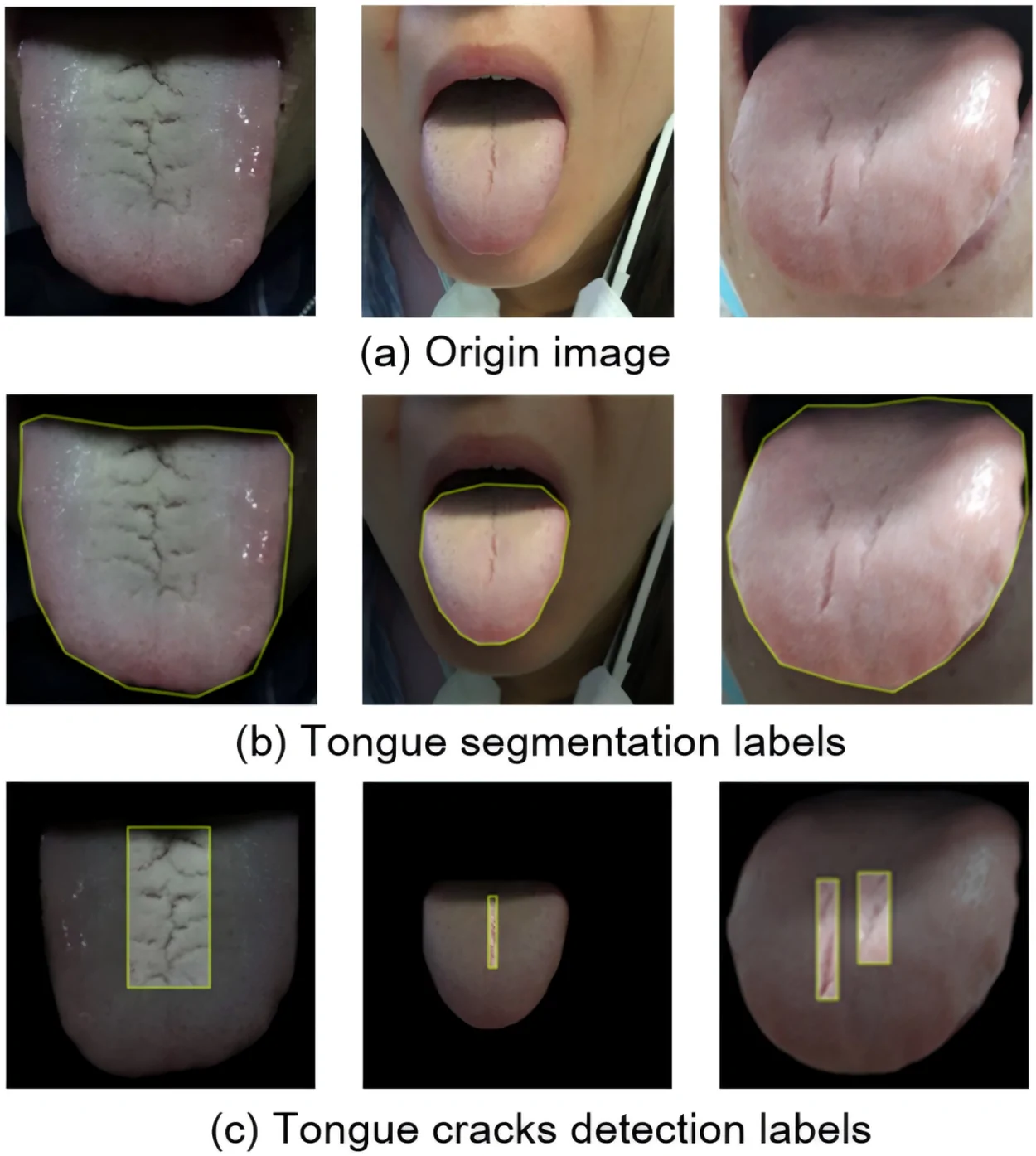
Example of the Tongue Image Dataset. **(a)** Original image. **(b)** Tongue segmentation labels. **(c)** Tongue crack detection labels.

**Table 1:** Tongue image dataset statistics.

Task		Training set	Validation set	Test set	Total
Crack detection	Cracks	254	57	41	352
	Non-cracks	516	163	69	748
	Total	770	220	110	1,100
Tongue segmentation		499	142	74	715

Table 2 presents the performance metrics of the YOLOv5 algorithm in the task of tongue segmentation. The model exhibits commendable performance in this task, with the detection box achieving Average Precision (AP) values of 99.5% at an IoU threshold of 0.5 (AP@50) and 92.9% at an IoU threshold of 0.5:0.95 (AP@50:95). Similarly, the segmentation masks attain AP@50 and AP@50:95 values of 99.5% and 94.2%, respectively. This signifies that the model is capable of detecting and segmenting the tongue body region with a high degree of accuracy across various IoU thresholds. The application of this model in the preprocessing step of tongue body area segmentation cropping is demonstrated in Figure 9.

**Table 2:** Performance of YOLOv5 on tongue segmentation.

Method	AP@50/%	AP@50:95/%
Detection frames	99.5	92.9
Masks	99.5	94.2

**Figure 9:**
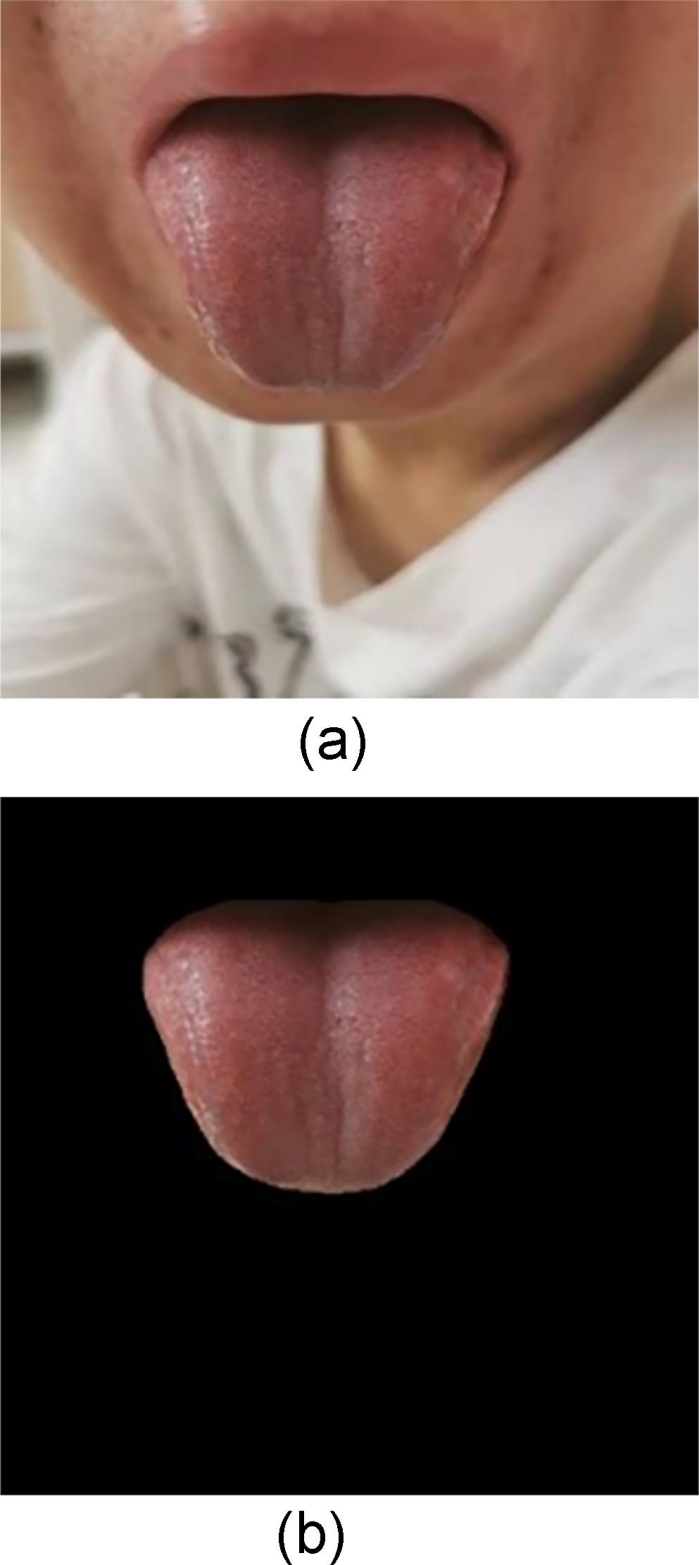
Tongue Segmentation and Cropping Preprocessing. **(a)** Original image. **(b)** Cropped image.

### 2.5 Experimental results

To rigorously validate the efficacy of our proposed Cracks-YOLO framework, we conducted a series of comprehensive experiments. This section delineates the quantitative and qualitative outcomes, commencing with an ablation study to dissect the individual contributions of the architectural enhancements, followed by a comparative analysis stateoftheart object detectors,and concluding with a visual interpretation of the model’s decisionmaking process.

#### 2.5.1 Ablation study on architectural components

We quantify the performance gains from Switchable Atrous Convolution (SAC) and the Transformer encoder via ablation experiments. Table 3 reports the results. The two modules exhibit a synergistic effect; their joint application improves model performance.

**Table 3:** Tongue cracks detection ablation experiment results.

SAC	Transformer	AP@50/%	AP@50:95/%	AP@75/%	AR@1/%	AR@10/%	AR@100/%	AUC-PR	PPV	NPV	Params (M)	GFLOPs
		83.53	60.49	69.12	60.23	67.21	68.84	91.31	94.29	90.14	**9.12**	**23.83**
✓		86.03	67.10	72.17	**69.07**	70.70	70.70	**98.32**	**100.00**	94.20	9.77	26.66
** ✓ **	** ✓ **	**86.60**	**69.80**	**72.27**	68.14	**73.26**	**73.26**	96.02	94.44	**94.44**	9.77	26.83

The bolded text represents the value that performs best in the corresponding column entry.

Integration of the SAC module into the backbone network improves performance over the baseline YOLOv5 model. Table 3 shows that the SAC-enhanced model raises AP@50 from 83.5% to 86.0% and AP@50:95 from 60.4% to 67.1%. This 6.71 percentage point increase indicates improved localization accuracy, enabling more accurate delineation of fine and irregular tongue fissure boundaries. This improvement originates from the adaptive receptive field mechanism of SAC. This mechanism allows the network to dynamically focus on multi-scale fissure features without substantially increasing parameter count, which rises from 9.12M to 9.77M.

After SAC incorporation, the Transformer encoder module is integrated into the deepest layers of the backbone and neck networks, further enhancing the model’s representational capacity. The complete Cracks-YOLO model, with both modules, achieves AP@50 of 86.6% and AP@50:95 of 69.8%. Relative to the baseline, these represent increases of 3.07 and 9.31 percentage points, respectively. The larger gain in AP@50:95 indicates improved performance in detecting cracks with precise spatial correspondence. The global receptive field of Transformer compensates for CNN’s inherent locality by modeling long-range dependencies and global contextual associations. This enables the model to distinguish fissures from visual distractors such as deep furrows or shadows. The performance gain is accompanied by a computational cost increase from 23.83 to 26.83 GFLOPs, meeting the real-time requirements of clinical decision support systems.

#### 2.5.2 Comparative evaluation with state-of-the-art detectors

We conduct a comparative evaluation against a range of established object detection frameworks. These frameworks cover both two-stage and one-stage architectures. Results in Table 4 indicate that Cracks-YOLO outperforms these frameworks.

**Table 4:** Model performance comparison in tongue crack detection.

Model	AP50/%	AP50:95/%	AP75/%	AR1/%	AR10/%	AR100/%	AUC-PR	PPV	NPV	Params (M)	GFLOPs
Faster R-CNN R50	81.64	69.38	72.86	70.70	71.16	71.16	98.04	89.74	**96.72**	41.30	267.85
FCOS R50	78.85	64.87	63.49	67.67	68.84	68.84	96.77	94.12	92.45	32.06	250.94
Mask R-CNN R50	84.08	68.90	70.59	68.37	70.93	70.93	97.60	89.74	95.83	43.92	267.85
RetinaNet R50	81.35	66.98	71.63	**72.09**	72.56	72.56	94.34	94.29	90.77	32.17	253.83
RT-DETR R50	67.59	52.29	59.32	61.16	63.72	67.21	80.86	83.33	95.45	42.76	130.47
SSD300 VGG16	65.97	57.57	58.26	61.63	62.09	62.09	94.42	96.43	85.92	23.75	60.86
SSDLite320 MobileNetV3	76.32	55.67	58.65	59.77	61.16	61.16	97.30	89.19	92.31	**2.21**	**0.89**
YOLOv10m	81.40	68.10	**72.90**	67.44	70.70	70.70	97.76	87.50	92.00	16.49	63.97
YOLOv10n	79.13	63.77	69.70	66.51	**73.49**	**73.49**	92.20	86.84	92.00	2.71	8.39
YOLOv10s	79.92	62.44	66.79	64.19	67.44	67.44	96.20	96.77	90.67	8.07	24.77
YOLOv3	84.93	67.90	68.51	66.98	71.63	71.63	96.28	97.06	95.83	103.69	282.98
YOLOv5s	83.53	60.49	69.12	60.23	67.21	68.84	91.31	94.29	90.14	9.12	23.83
YOLOv5s-SAC	86.03	67.10	72.17	69.07	70.70	70.70	**98.32**	**100.00**	94.20	9.77	26.66
**Cracks-YOLO (Proposed)**	**86.60**	**69.80**	72.27	68.14	73.26	73.26	96.02	94.44	94.44	9.77	26.83

The bolded text represents the value that performs best in the corresponding column entry.

Among all evaluated models, our model achieves the highest AP@50:95(69.80%). It outperforms detectors such as Faster R-CNN R50 (69.38%) and Mask R-CNN R50 (68.90%). Parameter count is reduced by over 75% (9.77M vs. 41.3M and 43.92M). Computational cost is reduced by a factor of ten (26.83 GFLOPs vs. over 260 GFLOPs). Under the more lenient AP@50 metric, Cracks-YOLO attains 86.60%, surpassing all other detectors except YOLOv5s-SAC. The higher AP@50 of YOLOv5s-SAC (86.03%) reflects the advantage of SAC. YOLOv10m yields a slightly higher AP@75 (72.90% vs. 72.27%) but requires substantially more GFLOPs (63.97 vs. 26.83). Its AP@50:95 is only 68.10%. This indicates the inefficiency of trading overall accuracy for a marginal threshold gain.

The recall values indicate the robustness of our detector. Cracks-YOLO achieves the highest AR@10and AR@100 (both 73.26%) among all models. This includes lightweight SDDLite MobileNetV3. It also includes powerful YOLOv10n. Our model consistently identifies a higher proportion of true fissures across varying detection confidence thresholds. This property is clinically significant. Missing subtle cracks is more detrimental than occasional false positives. The positive predictive value (94.44%) and negative predictive value (94.44%) are comparable to, or better than, most two-stage and one-stage models. These values reflect a balance between precision and recall.

The advantage over real-time detectors is more evident. SDDLite MobileNetV3 is a lightweight model with 2.21M parameters and 0.89 GFLOPs. Its AP@50:95 is 55.67%, a relatively low value. It fails to precisely identify fissures. The YOLOv10 series achieves higher efficiency. Its accuracies under the same metric are 62.44%, 63.77%, and 68.10%. YOLOv3 has 103.69M parameters and 282.98 GFLOPs. Its AP@50:95 is 67.90%. This indicates that merely increasing network depth is not the optimal solution for fine-grained detection tasks.

Our Cracks-YOLO model achieves the highest overall accuracy. It substantially outperforms other high-precision two-stage models in speed. This combination of high detection accuracy, low parameter count, moderate computational cost, and real-time processing capability demonstrates the practical feasibility of our framework in time-sensitive clinical decision support systems. The SAC module, with its adaptive multi-scale receptive fields, and the Transformer encoder, for global context reasoning, operate in synergy. The CSP-PAN neck network facilitates efficient multi-level feature fusion. This enables the detection system to achieve a balance between high precision and high efficiency. This is our primary advantage.

#### 2.5.3 Qualitative assessment and interpretability

To complement the quantitative results, we employed Gradient-weighted Class Activation Mapping (Grad-CAM) to visualize the discriminative regions that influenced the models’ predictions. As depicted in Figure 10, the improved YOLOv5 network exhibits a significantly more focused and precise activation pattern over the pathological crack regions compared to the baseline model. The original YOLOv5 often produces diffuse and scattered activations, indicating a less discriminative understanding of crack features. In contrast, our Cracks-YOLO demonstrates a highly localized and intense response precisely aligned with the fissures, confirming that the architectural enhancements have effectively sharpened the network’s attention.

**Figure 10:**
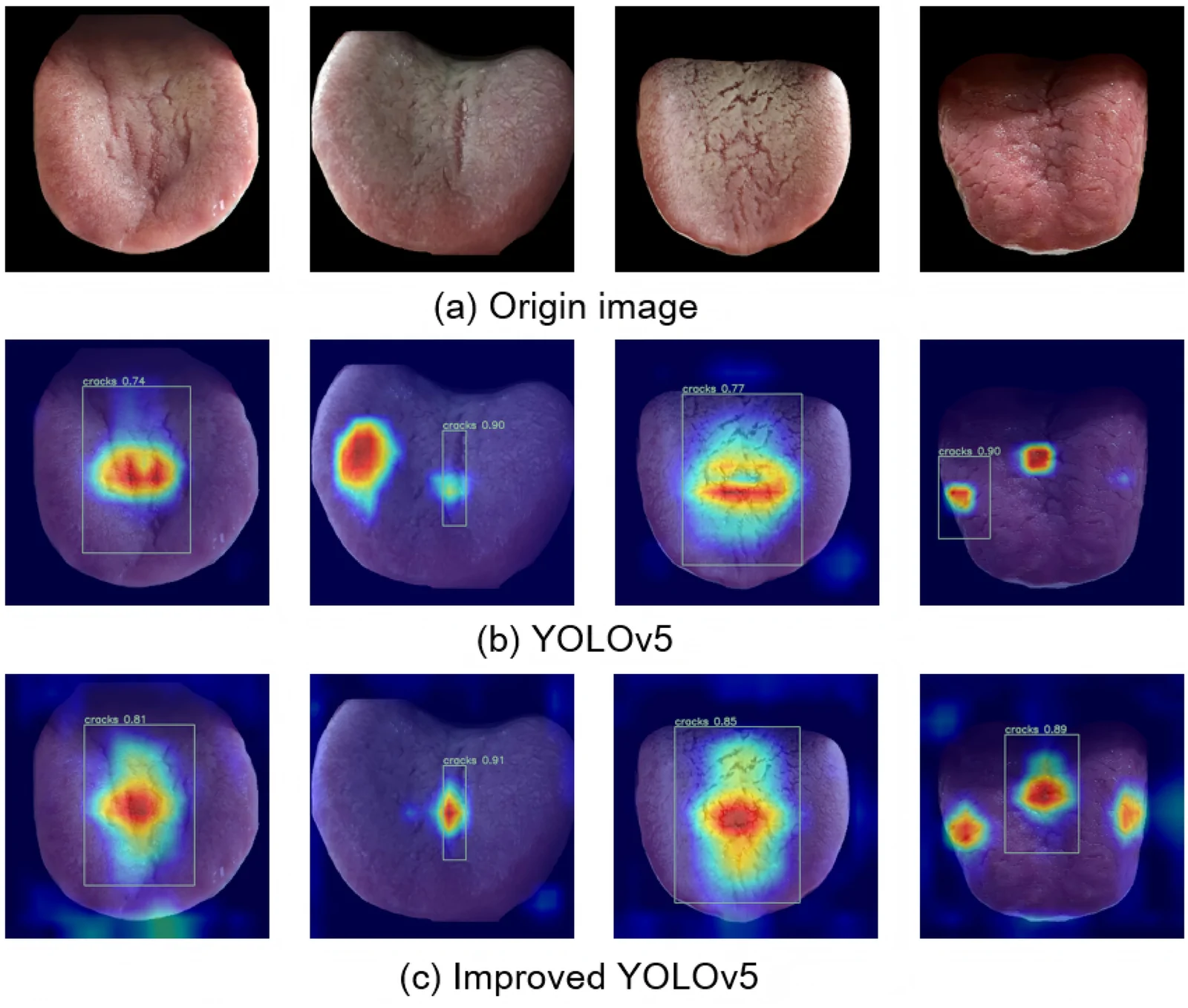
Grad-CAM visualization results. **(a)** Original image. **(b)** YOLOv5. **(c)** Improved YOLOv5.

Figure 10 illustrates the visualization results obtained using the Grad-CAM (42) which includes the detection outcomes from both the original YOLOv5 network and the improved YOLOv5 network. It is evident that, in comparison to the original network, the enhanced network places greater emphasis on and more distinctly highlights the crack regions, indicating that the improved network has a more accurate comprehension of the characteristics of tongue cracks. This outcome further substantiates the efficacy of the improved network in the detection of cracks.

This study has selected a subset of representative tongue crack data, and the test results for these samples are displayed in Figure 11. The tongue bodies in these test samples exhibit a variety of angles, colors, and levels of clarity, and the cracks themselves vary in morphology, distribution, and size. Despite the challenges posed by the diverse tongue characteristics and the variations in crack presentation, the model proposed in this study is capable of consistently detecting the location of cracks. This demonstrates that the model possesses a degree of robustness, enabling it to accommodate different tongue surface conditions and yield reliable detection outcomes.

**Figure 11:**
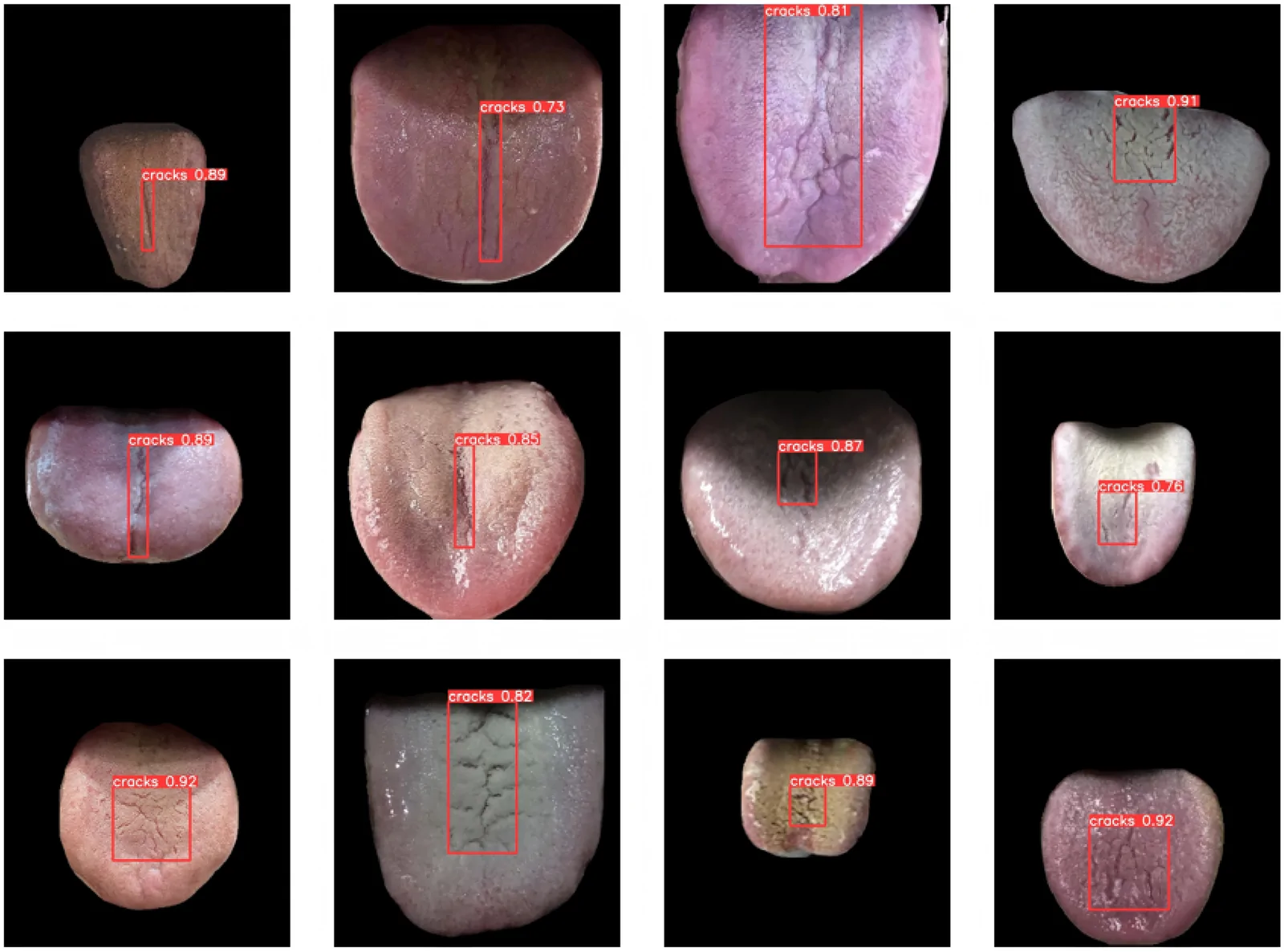
Examples of the testing results.

## 3 Conclusion

The development of objective tongue image analysis remains an important interdisciplinary research direction at the intersection of computer vision, medical image computing, and intelligent diagnostic support. This study proposes a deep learning-based tongue crack detection framework that improves YOLOv5 by integrating Switchable Atrous Convolution for adaptive multi-scale feature extraction and a Transformer encoder for global contextual modeling. The proposed design provides an effective solution for detecting subtle, irregular, and spatially diverse crack patterns while maintaining real-time inference efficiency. Future research may further expand this framework toward multi-task learning, instance-level crack segmentation, weakly supervised annotation strategies, domain adaptation across imaging devices, and multimodal clinical correlation analysis. With continued advances in deep neural architectures and visual representation learning, automated tongue image analysis is expected to evolve from isolated lesion detection toward a more robust, interpretable, and comprehensive computer-aided assessment system.

In extrapolating the applicability of Cracks-YOLO to authentic clinical practice, it is incumbent upon us to critically juxtapose several intertwined facets of generalizability—namely, photometric and hardware-induced variability, the inherent trade-off between model expressivity and finite training samples, and the demographic and institutional confines of our data provenance. While our proprietary dataset was deliberately curated to encompass appreciable internal diversity across illumination intensities, capturing sensors, and acquisition angles, its single-center origin inevitably circumscribes the extrapolative scope of our quantitative findings. We contend that the architectural innovations proposed herein—particularly the SAC-mediated dynamic multi-scale geometric sensitivity and the Transformer-imbued global contextual prior—collectively constitute a potent dual regularizer. This mechanism theoretically decouples lesion semantics from superficial colorimetric artifacts and mitigates overfitting to spurious local textures, a conjecture empirically substantiated by the marginal performance gap between training and validation splits, alongside the coherent morphological activations observed in Grad-CAM visualizations. We acknowledge that such theoretical resilience does not obviate the indispensable necessity for rigorous external validation against extreme color temperature shifts, unencountered imaging hardware, and disparate patient demographics—an uncharted frontier in the current study. Accordingly, we position this work as a robust foundational benchmark that substantiates the feasibility of hybrid CNN-Transformer paradigms for subtle lesion detection in Traditional Chinese Medicine. Our prospective roadmap unequivocally prioritizes the establishment of multi-center data-sharing consortia, complemented by the deployment of unsupervised domain adaptation, adversarial chromatic augmentation, and semi-supervised learning paradigms, to systematically dismantle the data-scarcity bottleneck and progressively translate these algorithmic advancements into dependable clinical decision-support instruments. As evidenced by the recent advances of Ju et al. (43, 44), these achievements collectively underscore the growing synergy between computational innovation and the translation of clinical applications in medical image analysis.

Nevertheless, several limitations remain in the current study. From an image-analysis perspective, crack-like patterns on the tongue surface may arise from different visual sources, including congenital fissures, fur-covered grooves, illumination variations, and pathological cracks. These visually similar structures introduce intra-class ambiguity and may lead to false-positive detections, particularly when crack boundaries are partially occluded or weakly contrasted against the surrounding tongue texture. Therefore, future work should incorporate more fine-grained annotation protocols, hierarchical crack categories, and auxiliary visual cues to improve the discriminative capability of the detector. Moreover, the present study focuses primarily on tongue crack localization, whereas a more comprehensive computational analysis of tongue images requires the joint modeling of multiple morphological and textural attributes, such as tooth marks, spots, tongue-body thickness, coating distribution, and surface color. Extending the proposed framework from single-target crack detection to multi-attribute tongue image understanding would further enhance its applicability in automated tongue image analysis. In addition, richer crack-level descriptors, including orientation, shape, depth-related appearance, spatial distribution, and anatomical location, could be quantitatively extracted after detection. These structured visual features may provide more informative representations for downstream risk assessment, longitudinal monitoring, and non-contact screening applications.

## Data Availability

The original contributions presented in the study are included in the article/supplementary material, further inquiries can be directed to the corresponding author.
